# Diagnosis of a model of Duchenne muscular dystrophy in blood serum of *mdx* mice using Raman hyperspectroscopy

**DOI:** 10.1038/s41598-020-68598-8

**Published:** 2020-07-16

**Authors:** Nicole M. Ralbovsky, Paromita Dey, Andrew Galfano, Bijan K. Dey, Igor K. Lednev

**Affiliations:** 1 Department of Chemistry, University At Albany, SUNY, 1400 Washington Avenue, Albany, NY 12222 USA; 2 The RNA Institute, University At Albany, SUNY, 1400 Washington Avenue, Albany, NY 12222 USA; 3 Department of Biological Sciences, University At Albany, SUNY, 1400 Washington Avenue, Albany, NY 12222 USA

**Keywords:** Diseases, Diagnostic markers, Raman spectroscopy

## Abstract

Duchenne muscular dystrophy (DMD) is the most common and severe form of muscular dystrophy and affects boys in infancy or early childhood. Current methods for diagnosing DMD are often laborious, expensive, invasive, and typically diagnose the disease late in its progression. In an effort to improve the accuracy and ease of diagnosis, this study focused on developing a novel method for diagnosing DMD which combines Raman hyperspectroscopic analysis of blood serum with advanced statistical analysis. Partial least squares discriminant analysis was applied to the spectral dataset acquired from blood serum of a mouse model of Duchenne muscular dystrophy (*mdx*) and control mice. Cross-validation showed 95.2% sensitivity and 94.6% specificity for identifying diseased spectra. These results were verified via external validation, which achieved 100% successful classification accuracy at the donor level. This proof-of-concept study presents Raman hyperspectroscopic analysis of blood serum as an easy, fast, non-expensive, and minimally invasive detection method for distinguishing control and *mdx* model mice, with a strong potential for clinical diagnosis of DMD.

## Introduction

Duchenne muscular dystrophy (DMD) is a progressive form of muscular dystrophy which typically affects male infants. DMD is an X-chromosome linked recessive disorder caused by a loss of function of the dystrophin gene of 2.3 million base pairs, which results in progressive weakness and atrophy of the skeletal and cardiac muscles.^1,2^ The issues associated with DMD are severe, worsen overtime, and greatly impact the well-being of the afflicted individual. In fact, secondary complications due to DMD, including cardiac and respiratory muscle problems, can lead to life-threatening conditions.^3^ Although there is no cure, limited treatment regimens exist for DMD which can slow the progression of the symptoms associated with the disease.

Diagnosing DMD typically involves evaluating family history as well as conducting blood tests to assess the levels of specific muscle enzymes in the blood. Although the inheritance of the disease is through an X-linked recessive pattern, there are cases where DMD occurs in families who have no history of it. The complicated pattern of inheriting DMD suggests a need for additional testing. Blood tests often monitor the level of serum creatine phosphokinase (CPK), however, this test can only detect the disease in later stages and is generally non-specific, as high levels of CPK can be found in an individual’s blood after experiencing a heart attack, drinking alcohol in excess, or participating in strenuous exercise.^4–9^ Electromyography can confirm muscle weakness without pinpointing a direct cause of it.^10^ Muscle biopsies can differentiate muscular dystrophies from other muscle diseases,^11^ however biopsy examinations can be both expensive and invasive. Further, biopsies and genetic testing are typically pursued only after other options have been exhausted, resulting in the disease being diagnosed in its later stages. Because DMD is progressive, it is of the utmost importance to definitively diagnose the disease as early on in its progression as possible. The earlier the disease is identified, the better opportunity the afflicted individual has for seeking treatment opportunities to slow the progression of the disease phenotype.

To improve the accuracy, ease, and potential of an early diagnosis, we focused on developing a novel method for diagnosing DMD using Raman hyperspectroscopic analysis of *mdx* mouse blood serum combined with advanced statistical analysis. Most DMD patients display deletion mutations of one or more of the 79 exons in the DMD gene, leading to out-of-frame mutations and loss of dystrophin protein in their muscle fibers.^12^ Similar to patients, the dystrophin mutant *mdx* mice do not express dystrophin^13^ and have been widely used as a model system to study DMD and to make important advances in understanding therapeutic strategies as well as the molecular processes and underlying causes of the disease.^2,14^ The *mdx* mouse model serves as an efficient model for developing a better diagnostic method without influence from complications, such as the effect of prescribed medications, associated with humans.

Raman hyperspectroscopy has shown great potential to diagnose many diseases including cancers,^15,16^ Alzheimer’s disease,^17–19^ and others.^20,21^ Raman hyperspectroscopy involves collecting multiple Raman spectra from a sample to better characterize its inherent heterogeneity and understand its biochemical composition. This allows for the detection of changes in biological composition of blood serum due to disease progression. Because Raman hyperspectroscopy produces this specific spectral fingerprint for each sample, different samples can be distinguished, including dried traces of body fluids collected from healthy donors and from donors with a disease. Here, we capitalized on the advantages of Raman hyperspectroscopy in combination with advanced statistical analysis to build a model which identifies spectral differences between different classes of samples to make diagnostic predictions. Partial least squares discriminant analysis (PLS-DA) was used to build a model which could distinguish Raman spectral data of healthy control mice from Raman spectral data of *mdx* mice. The results were verified using external validation. Genetic algorithm (GA) identified spectral features which contribute the most useful information toward differentiation; these features were assigned to vibrational modes of various biomolecules previously identified as playing a role in the pathogenesis of DMD. For the first time, this proof-of-concept study shows Raman hyperspectroscopy in combination with advanced statistical analysis is successful in distinguishing control from *mdx* model mice in a simple, accurate, early, and minimally invasive manner, indicating a strong potential for clinical diagnosis of DMD.

## Results

### Validation of skeletal muscle abnormalities in *mdx* mice by examining the tibialis anterior (TA) muscle morphology

**Figure 1:**
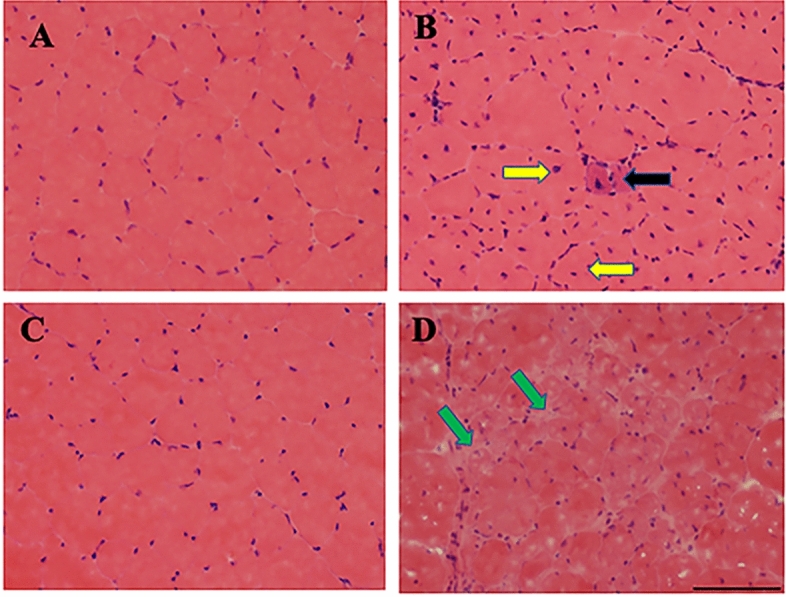
Skeletal muscle degeneration is observed in the mouse model of DMD. Hematoxylin and Eosin (H&E) staining of TA muscle cross sections from 3- and 12-month-old control (C57BL/10ScSnJ) (**A**, **C**) and *mdx* (C57BL/10ScSn-Dmd < mdx > /J) (**B**, **D**) mice. The 3-month old control muscle cross-section shows normal fiber morphology including circular shape and absent central nuclei (**A**), whereas 3-month old *mdx* mice show muscle degeneration denoted by muscle fibers with central nuclei observed and smaller diameter (yellow arrows), atrophied muscle (smaller than the neighboring fibers without central nuclei, black arrow), and more prevalent nuclei which represents inflammatory cells (**B**). Control mice at 12-months old (**C**) are compared to the 12-month old *mdx* mice (**D**) where muscle degeneration is much more dramatic, as evident by the absence of normal muscle fiber structure in almost all areas of the section; the muscle structure is often taken over by fatty and necrotic tissues, as indicated by the unstained/white areas (green arrows). Scale Bar: 100 µM.

Duchenne muscular dystrophy is the most common and most severe form of muscular dystrophy. DMD is characterized by muscle wasting and weakness due to excessive muscle degeneration. The tibialis anterior (TA) muscle morphology of 3-month old and 12-month old control (C57BL/10ScSnJ) and *mdx* (C57BL/10ScSn-Dmd < mdx > /J) mice was examined using Hematoxylin and Eosin (H&E) staining (Fig. 1A–D). As expected, normal skeletal muscle morphology was observed in 3-month old control mice (Fig. 1A). Mild skeletal muscle degeneration was observed in 3-month old *mdx* mice as characterized by the smaller diameter of muscle fibers with central nuclei, occasional presence of atrophied muscle fiber, and the presence of an increased number of nuclei, representing inflammatory cells (Fig. 1B). Similar to 3-month old control mice, 12-month old control mice displayed normal skeletal muscle morphology (Fig. 1C). Skeletal muscle degeneration progresses as *mdx* mice get older. As such, muscle degeneration was much more prominent in the 12-month old *mdx* mice as marked by the absence of normal muscle structure in most areas of the tissue section and the presence of fatty and necrotic tissues (Fig. 1D).

### Raman spectroscopic analysis of mice blood serum

Because DMD is progressive, it is crucial to develop a simple diagnostic tool for identifying the disease as early as possible. In this proof-of-concept study, dried blood serum of healthy and *mdx* mice at 3- and 12-months old was analyzed by Raman hyperspectroscopy in an attempt to develop a novel diagnostic method. Blood serum is the portion of blood which does not contain cells or clotting factors, and has been widely studied in the past for diagnostic purposes.^18,22–25^.

**Figure 2:**
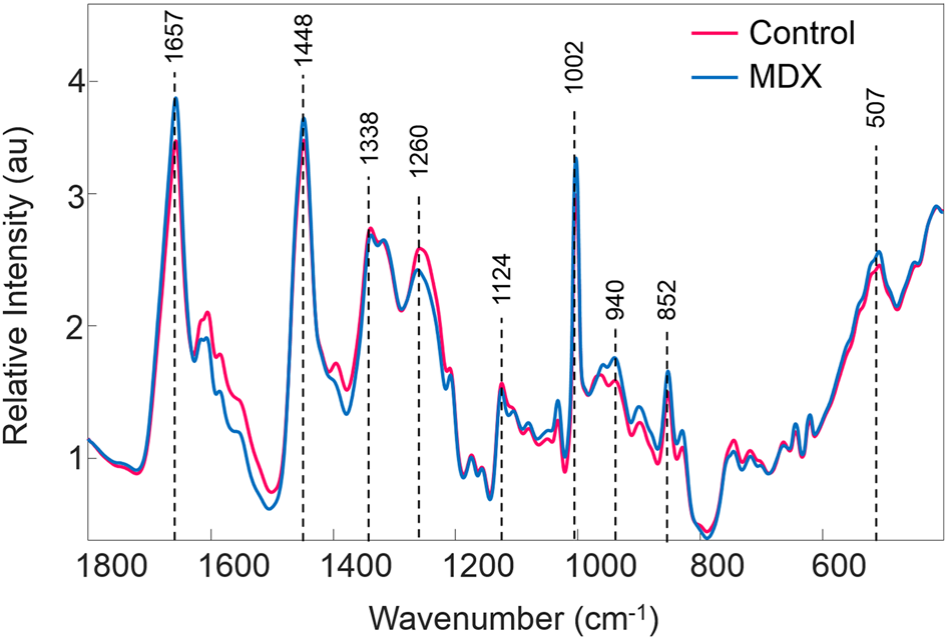
Mean preprocessed Raman spectra collected from the two classes of dried mice blood serum. The mean preprocessed spectrum of all control mice blood serum samples is represented by the pink line, whereas the mean preprocessed spectrum of all *mdx* mice blood serum samples is represented by the blue line.

Raman spectra were collected from the serum of 14 mice donors through automatic mapping. Mapping was conducted to obtain an accurate representation of the entire biochemical composition of each dried serum sample, with the intention of identifying key biochemical components useful for discrimination between classes. The two classes of donors consisted of healthy mice (control, n = 7) and *mdx* model mice (MDX, n = 7). Of the 14 total blood serum samples, six (three control and three MDX) were collected from mice at three months old and eight (four control and four MDX) were collected from mice at 12 months old. The three month old *mdx* mice are considered a model of early DMD, and 12 month old *mdx* mice are considered a model of late stage DMD. The mean preprocessed spectra for all donors from each class is seen in Fig. 2. Peak assignments are discussed further on.

### Model calibration for differentiating healthy controls from MDX mice

The donors were split into two groups: the calibration group and the validation group. The spectral data from the ten donors of the calibration set (five control, five MDX) was used to build the PLS-DA prediction algorithm. The spectral data from the validation dataset, consisting of two control and two MDX donors, were set aside and used for external validation. Mice of different ages (3- and 12-months) were included in both groups.

The difference between the mean control and the mean MDX spectrum was calculated and compared with ± 2 standard deviations within each class. The difference spectrum fell within the standard deviations (Supplementary Fig. S1), indicating that the spectral changes shown in the difference spectrum are smaller than the variation which occurs within each class, and are statistically insignificant. Advanced statistical analysis is thus required to capitalize on the important spectral features which vary between the two classes at the level of individual spectra but are hidden from the mean spectra. This variability is useful for discriminating between the two classes of data.

**Figure 3:**
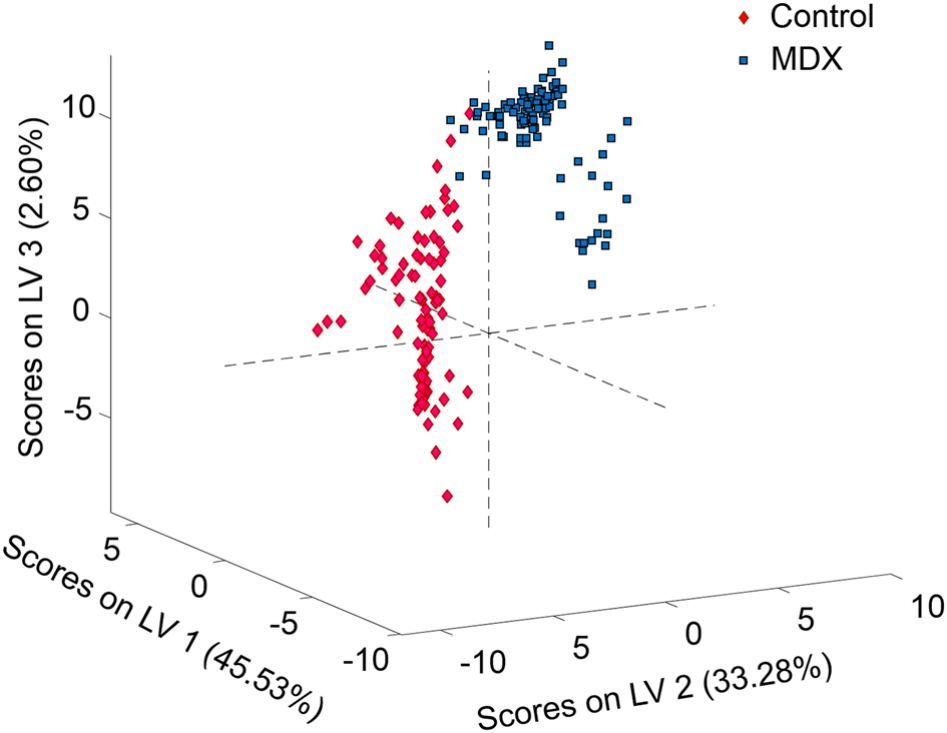
PLS-DA scores plot. The PLS-DA scores plot built using the first three latent variables. The distribution of symbols represents the separation which exists between the two classes of blood serum spectra where pink diamonds signify controls and blue squares signify MDX. Each symbol represents an individual spectrum.

Partial least squares discriminant analysis (PLS-DA) was selected to build a discrimination algorithm. A binary model was built to distinguish between control and MDX blood serum spectral data of the calibration dataset. Eight latent variables captured the maximum covariance between the spectral data and the assigned classes. Each spectrum from the calibration dataset was assigned a set of scores which correspond to how similar that spectrum is to each latent variable. Each class is ideally represented by a range of scores seen as typical for that class. Scores plots can be used to understand the separation which exists between different classes, and any spectrum which is loaded into the model will be given a set of scores which is used to decide to which class it belongs. The model built herein showed clear separation between the two classes (Fig. 3).

The sensitivity and specificity rates for classification of the PLS-DA diagnostic algorithm were calculated. In this study, the sensitivity is defined as the true positive rate, or percentage of MDX spectra correctly predicted as belonging to the MDX class. The specificity is defined as the true negative rate, or percentage of control spectra correctly predicted as not belonging to the MDX class. Individual spectral predictions for all donors within the calibration dataset are observed in the confusion matrix presented in Table 1. Here, every Raman spectrum is assigned a class (either control or MDX). The assignments are compared to the true, or known, classification for each spectrum. Cross-validation of the PLS-DA model by venetian blinds resulted in 95.2% sensitivity and 94.6% specificity for training the algorithm using the calibration dataset.

**Table 1:** Cross-validated (CV) and external validation (ext. val.) PLS-DA prediction results for distinguishing between control and MDX blood serum donors.

Model parameters	Control	MDX
Sensitivity		
CV	0.94643	0.95175
Ext. val	0.86957	1.00000
Specificity		
CV	0.95175	0.94643
Ext. val	1.00000	0.86957
Predicted as control		
CV	212	11
Ext. val	80	0
Predicted as MDX		
CV	12	217
Ext. val	12	93

### External validation of the PLS-DA model

External validation was performed using the spectral data collected from the four donors of the validation dataset. The validation dataset was kept independent from the training set and is considered a powerful method for testing the validity and strength of the classification model. A total of 185 spectra collected from the four samples were loaded into the PLS-DA algorithm for external validation. The class assignment for each spectrum was predicted (Table 1). Again, the sensitivity and specificity of classification for external validation were calculated. Here, 100% sensitivity and 87.0% specificity was achieved for external validation at the level of individual spectral predictions.

### Receiver operating characteristic curve analysis of external validation results

**Figure 4:**
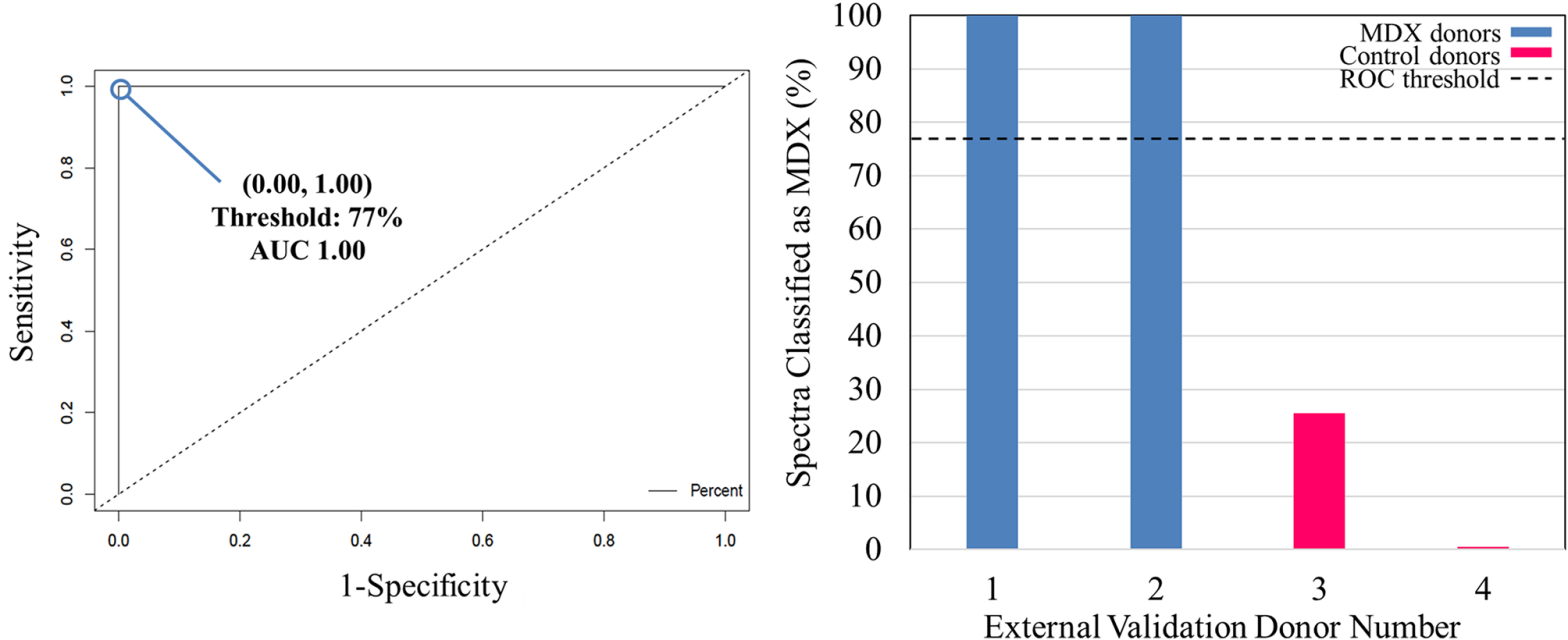
Receiver operating characteristic (ROC) curve and PLS-DA external validation results. (**A**) ROC curve for the cross-validated PLS-DA model, trained to differentiate between diseased and healthy control mice blood serum. The true positive rate (sensitivity) of each potential discrimination threshold are plotted according to each corresponding false positive rate (1—specificity). The optimal threshold is designated by the point at (0.00, 1.00), corresponding to a threshold of 77%. (**B**) The percentage of spectra classified as MDX is plotted as the bar height of each of the donors. The 77% threshold is plotted as the dashed line.

A receiver operating characteristic (ROC) curve was used to identify the optimum threshold for determining donor-level classifications based on spectral-level predictions. A ROC curve evaluates the performance of a binary classifier and is generated by plotting true positive rate values (sensitivity) against false positive rates values (1-specificty). Every point on the ROC curve corresponds to a potential threshold for discrimination. The ROC curve generated for the PLS-DA model built in this study, based on cross-validation, is seen in Fig. 4A. The most optimum threshold for discrimination in this study is designated by the point at (0.00, 1.00), which corresponds to a cut-off value of 77%. This threshold indicates if 77% or more of the total spectra from a donor in the external validation dataset are assigned to the MDX class, than the overall prediction of the donor would be as belonging to the MDX class.

The threshold established by the ROC curve (77%) was applied to the model’s spectral-level predictions to generate a diagnosis at the donor level for external validation, as shown in Fig. 4B. The percentage of spectra which were identified as belonging to the MDX class is plotted as the height of the bar. The 77% threshold is then applied to each of the four donors. External validation donors 1 and 2 were found to have greater than 77% of their total spectra predicted as being MDX, and thus the overall donor was predicted as belonging to the MDX class; the opposite was true for external validation donors 3 and 4. Upon comparing these donor-level predictions with the true diagnosis of each, it was found that all four donors in the validation dataset were correctly identified. Thus, based on donor-level predictions, 100% successful external validation was achieved. This indicates the strength and capability of the model to be applied to new, unknown data, to make accurate diagnoses.

### Genetic algorithm for identifying spectral differences in blood serum

**Figure 5:**
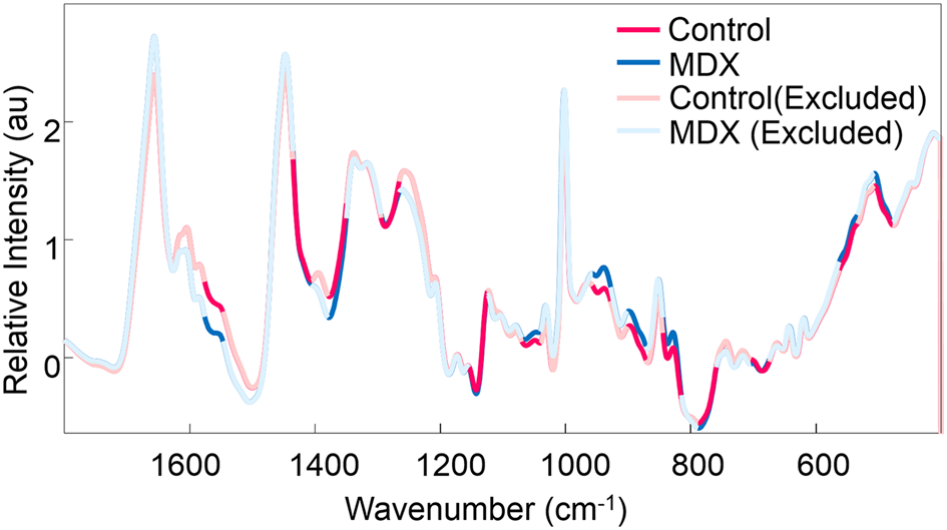
Genetic Algorithm analysis. Mean preprocessed dried blood serum spectra of the two classes, including the spectral ranges selected by Genetic Algorithm: control (pink) and MDX (blue). Areas selected by Genetic Algorithm are marked by bolded lines. Spectral regions deemed as uninformative for discrimination are seen as unfilled lines.

Genetic algorithm (GA) was performed to better understand the biochemical basis responsible for the model’s ability to discriminate between spectral datasets. GA is a statistical technique which capitalizes on the ideas of “natural selection” and “survival of the fittest.”^26^ The algorithm identifies spectral features within the dataset which contribute the most discrimination power toward separating classes of data and further provides insight into the biochemical changes that occur as the disease progresses. The results of GA are observed in Fig. 5. The tentative assignments of the Raman bands identified by GA can be attributed to various biomarkers which have been previously shown to be linked to DMD; these are summarized in Table 2.

**Table 2:** Tentative assignments of the most important regions in the Raman spectrum of blood serum for discrimination between control and MDX mice, as determined by GA.

	GA region	Peak position (cm^-1^)	Vibrational mode	Contributions^65–69^
1	479–507	507	(S–S)^a^	Collagen; Cysteine
2	535–563	541	(S–S)^a^	Cysteine; Cholesterol
3	675–703	702		Cholesterol; Cholesterol Ester
4	760–786	750	Ring breathing mode	Pyrimidines of DNA/RNA bases
5	815–844	829	(O–P–O)^a^; out-of-plane ring breathing	DNA/RNA; Tyrosine
6	872–899	878	(C–C–N^+^)^b^	Lipids
		899	(C–O–C) skeletal mode	Monosaccharides; Disaccharides
7	929–960	940	Skeletal mode	Polysaccharides
		959		Cholesterol
8	1,042–1,066	1,048		Glycogen
9	1,124–1,156	1,124	(C–C)^a^; (C–N)^a^	Lipids; Proteins; Glucose
		1,156	(C–C)^a^	Proteins
10	1,260–1,294	1,260	Amide III	Proteins
11	1,340–1,377	1,338	(CH_2_/CH_3_)^c,d^	Collagen; Lipids
12	1547–1575	1554	Amide II	Proteins

^a^Stretching.

^b^Symmetric stretching.

^c^Wagging.

^d^Twisting.

## Discussion

The combination of Raman hyperspectroscopy and advanced statistical analysis is incredibly advantageous for disease diagnostic purposes. Raman hyperspectroscopy involves the collection of multiple Raman spectra from a sample to characterize its heterogeneity. This is accomplished through acquiring spectral information alongside spatial information, allowing for the formation of a three dimensional data cube (*x*, *y*, *λ*). Two dimensions, *x* and *y*, correspond to spatial coordinates, and the third dimension, *λ*, represents the Raman spectrum collected at a particular pair of coordinates. By probing multiple small areas of a sample, there is a potential to identify biochemical components which, although may be present at low average concentrations, are present at a particular coordinate at a high local concentration. The ability to detect such components using this method indicates they may be useful for discrimination, and can serve as spectroscopic biomarkers. Thus, the advantage of Raman hyperspectroscopy resides in its ability to detect multiple biomarkers simultaneously, which can be used for discrimination and diagnostic purposes.^17^

It is often observed that spectral differences between two similar classes of samples, such as dried traces of healthy and diseased body fluids, are insignificant when evaluated at the average level.^18,27^ It is expected that the majority of the composition of a body fluid remains consistent between healthy and diseased donors. In this research, the difference spectrum calculated between the average control spectrum and the average MDX spectrum of dried serum was shown to be statistically insignificant. This indicates that statistical analysis is required to better understand and evaluate the Raman spectral data obtained, and specifically, to uncover hidden characteristic features of the two classes as well as spectral variability which can be capitalized on for building a discrimination algorithm. In this study, the combination of Raman hyperspectroscopy and advanced statistical analysis was used to develop an algorithm which could accurately distinguish between control and *mdx* model mice through analysis of dried blood serum.

The *mdx* mice model was specifically selected for this project because the species exhibits a mutation within its DMD gene, resulting in the mouse not expressing the dystrophin protein and developing the disease. This animal model has been widely studied in the last several decades, and has provided extensive insight into the pathophysiology associated with muscular dystrophy.^2,14^ Additionally, the *mdx* mouse model can be manipulated to test potential therapeutic strategies, and lack of interfering factors, such as comorbidities or influence of prescribed medications, makes it ideal for evaluating novel diagnostic methods.

PLS-DA was selected to generate the prediction algorithm. The 14 donors used in this study were split into two groups: a calibration set and a validation set. The spectral data from the calibration set, consisting of 452 total spectra from five control and five MDX donors, was used to build and train the prediction algorithm. Cross-validation by venetian blinds resulted in 95.2% sensitivity and 94.6% specificity for identifying MDX spectra.

The prediction capabilities of the algorithm was then tested through external validation using the validation set of samples, consisting of two control donors and two MDX donors. The spectral data from the validation dataset was used to test the ability of the algorithm to make predictions regarding samples it has never before seen, and thus cannot have an inherent bias toward. The PLS-DA algorithm generated classification predictions for each individual spectrum collected from the four donors. Each sample is represented by a multitude of spectra; because dried traces of blood serum are inherently heterogeneous,^28^ each spectrum is expected to deviate from the mean to some extent. It is also expected that a portion of the mice blood serum components are the same between control and *mdx* model donors. As such, it is reasonable to assume that some spectra from one class may be predicted as belonging to the other due to the natural overlap in biochemical composition. ROC curve analysis was used to establish an optimum threshold for donor-level predictions. Using the determined threshold of 77%, all four donors of the validation dataset were identified as belonging to their true class, resulting in 100% accuracy at the donor level. External validation is an established process for determining whether or not a model is robust enough for successful application to new and unknown spectral data for accurate predictions.^29,30^ Successful external validation, as achieved here, indicates the potential for the method to be applied within diagnostic settings.

The contribution of multiple biomarkers to the spectroscopic signature of DMD as determined by GA allows for much more specific identification of the disease, and further supports the strength of the method. In general, by identifying biochemical components whose alterations in composition or concentration reflect the presence of a particular disease, the ability to detect that disease is dramatically increased, and can result in very high levels of classification accuracy.^17^ Past literature has demonstrated strong links between the pathogenesis of DMD and the tentatively assigned biomolecules. Specifically, studies have shown that a general increase in lipids, including triglycerides, phospholipids, cholesterol, and cholesterol esters, is found in patients with muscular dystrophy.^31,32^ In fact, in *mdx* model mice, elevated lipid levels were found to be associated with significant exacerbation of muscle pathology, including myofiber damage and skeletal muscle remodeling.^32^ Collagen has also been found to play a role in the pathogenesis of muscular dystrophy.^33^ Among the evidence, researchers found an inverse relationship exists between the over-production of connective tissue and muscle protein synthesis in patients suffering from DMD.^34–36^ Other research observed unusual clusters of “sticky cells” formed by dissociated muscle of patients with Duchenne and Becker muscular dystrophies, a sign which reflects abnormal collagen production.^37^ Mutations in genes coding for collagen type VI are also responsible for congenital muscular dystrophies including Bethlem myopathy and Ullrich congenital muscular dystrophy.^38^.

Many serum proteins have been identified as biomarkers which reflect the pathogenesis of DMD; the concentration of 23 identified mouse serum proteins exhibited an increase while four other proteins were found to exist at concentrations significantly lower in *mdx* model mice as compared to healthy control mice in one study. Proteins which were elevated mostly originated from muscle or were glycolytic enzymes, transport proteins, or other proteins such as creatine kinase M.^39^ These identified protein biomarkers reflect the muscle activity as well as pathogenesis of the disease. Many more studies have also identified various serum proteins as biomarkers for muscular dystrophy.^40–43^ It is thus unsurprising that GA identified spectral features which can be attributed to vibrational modes of proteins as being useful for discrimination purposes. Furthermore, a relationship between glycogen metabolism and DMD was supported by Naim et al. Here, results show that *mdx* model mice have increased skeletal muscle glycogen content; many of the enzymes involved in the skeletal muscle glycogen metabolism were dysregulated.^44^ Because of the dysregulation of glycogen, levels of glucose in the blood may be affected, connecting the identification of both glycogen and glucose here as also being important spectroscopic markers for DMD.

Notably, the spectral features identified by GA as being the most useful for spectroscopically discriminating between the two classes of data can also be assigned to vibrational modes of classes of biomolecules which have previously been related to the pathogenesis of the disease itself. Clearly, there is a connection between the progression of the disease and the spectroscopic signature produced. This link is strong enough to provide identifiable information which can be capitalized on through advanced statistical analysis for the purpose of generating a successful diagnostic algorithm and through the identification of the aforementioned biomolecules associated with DMD, we were indeed able to achieve high levels of diagnostic accuracy. Raman hyperspectroscopy allows for simultaneous detection of multiple, potentially new, biomarkers for a disease. This is incredibly advantageous over other diagnostic methods which simply investigate one, known, biomarker at a time.

## Conclusion

The method of combining Raman hyperspectroscopy with advanced statistical analysis is shown in this proof-of-concept study to be successful for distinguishing control and *mdx* model mice, with a substantial potential for clinical detection of Duchenne muscular dystrophy. Raman spectra were collected from traces of blood serum from either healthy control or diseased mice. The spectral data was analyzed using PLS-DA, which showed 95.2% sensitivity and 94.6% specificity for identifying MDX spectra in the calibration dataset, and 100% sensitivity and 87.0% specificity for identifying MDX spectra in the validation dataset. Based on donor-level predictions generated using ROC curve analysis, 100% accuracy was achieved for correctly predicting to which class the donors in the external validation dataset belonged. This is the first time this methodology has been applied toward distinguishing control and *mdx* model mice for the purpose of identifying DMD. Genetic Algorithm identified key biochemical components which were responsible for spectroscopic discrimination, indicating a link between the disease progression and the Raman spectroscopic fingerprint. Future research is required to study this link on a larger scale, and to investigate if a similar trend is observed within humans. It is clear that this methodology has significant potential for use as a novel technique for diagnosing Duchenne muscular dystrophy in clinical settings.

## Methods

All experimental protocols were approved by the Institutional Animal Care and Use Committee and the Laboratory Animal Resources Standard Operating Procedures; all methods were carried out in accordance with relevant guidelines and regulations.

### Mouse strains and sample collection

The *mdx* (C57BL/10ScSn-Dmd < mdx > /J; Stock Number 001801) and counterpart control mice (C57BL/10ScSnJ; Stock Number 000476) were purchased from the Jackson Laboratory, Bar Harbor, ME, USA. The mice were raised following the protocol approved by the Institutional Animal Care and Use Committee to the appropriate age (3 months and 12 months) before harvesting the tissue and blood samples. As Duchene muscular dystrophy is an X-linked muscle degenerative disease, male *mdx* and male control mice were studied. 3-month-old mice are equivalent to young adult humans and 12-month-old mice are equivalent to adult humans. However, though the *mdx* mouse contains only a single mutation on exon 23 of the DMD gene, the phenotypes of 3-month-old mice is considered equivalent to early DMD phenotypes in patients.^45^.

Mice were euthanized following the standard operating procedure of Laboratory Animal Resources (LAR SOP # 105 and 106). Briefly, the mice were first anesthetized to a surgical plane of anesthesia under isoflurane inhalation using an induction chamber. The depth of anesthesia was verified by establishing the loss of pedal reflex. The mice were euthanized under anesthesia by isoflurane and then by cervical dislocation. For harvesting skeletal muscle, the hind leg skins were removed and the Tibialis Anterior (TA) muscles were removed by a surgical blade. The TA muscles were cut into 2 pieces and frozen fresh with Optimal Cutting Temperature (OCT) compound in plastic molds. The freezing process was carried out in a jar containing semi-frozen iso-butanol and again frozen in liquid nitrogen before storing the tissue blocks at − 80 °C. The blood samples were collected from the euthanized mice by cardiac puncture. Briefly, the skin and the rib cases were cut and pinned in the dissection board. The jugular vein was cut by sharp scissors and blood was collected in small Eppendorf tubes, without use of anticoagulant, using pasteur pipettes.

### Isolation of serum

The serum was isolated following a standard laboratory protocol. Briefly, the tubes containing the blood without any anticoagulant were left at room temperature in a standing position for about 35 min, allowing the blood to clot. Then, the clotted blood samples were centrifuged at 20 °C and 2000*g* for 15 min; the serum fraction was moved to a fresh tube and stored at − 80 °C. At the time of analysis, the blood serum was allowed to thaw. Each serum sample (10 µL) was deposited on an aluminum foil substrate and set aside to dry overnight before analysis.

### Cryosection and histochemistry of TA muscle

The cryosections and H&E staining was carried out using established protocol as described elsewhere.^46,47^

### Raman hyperspectroscopy

A Renishaw inVia Raman spectrometer equipped with a research-grade Leica microscope was used to collect Raman spectra of dried blood serum. A PRIOR automatic mapping stage was used during measurements and the 50X objective was used to focus on the sample. Spectra were recorded between 400 and 1,800 cm^−1^ under excitation by the 785 nm diode laser, which was reduced to about 50% laser power to prevent photo-degradation of the sample. For each sample, 50 spectra were recorded to capture the inherent heterogeneity of the dried blood serum.

### Data treatment and advanced statistical analysis

Spectra were recorded using WiRE 3.2 software, and then imported to PLS_Toolbox (Eigenvector Research Inc.) which operates within MATLAB version 2017b software (Mathworks, Inc.). Any individual Raman spectrum which displayed a poor signal-to-noise ratio or exhibited cosmic rays was removed from the dataset. The remaining spectra were subjected to preprocessing, including baseline correction, normalization, and mean centering methods available within PLS_Toolbox, before performing analysis. Tentative peak assignments were made (after applying Genetic Algorithm) and are summarized in Table 2.

### Partial least squares discriminant analysis (PLS-DA)

PLS_Toolbox (Eigenvector Research, Inc.) was used for statistical analysis. PLS-DA was selected to accomplish discrimination between the healthy and diseased classes. PLS-DA algorithms have been shown to be effective in various disease diagnostic applications including for investigating inflammatory bowel diseases,^48^ coronary heart diseases,^49^ and various forms of cancer,^50–62^ among many others. Specifically, PLS-DA is a supervised technique which is used to predict categorical variables. The dataset being analyzed is reduced to a few latent variables (LVs), which capture the maximum covariance between spectral data and the labeled classes. Each spectrum is then given a score which corresponds to how closely that spectrum resembles a particular LV. Different classes of samples will be represented by a set of scores seen as characteristic for a sample within that class.^63^ In this way, unknown samples can be identified through comparison of the unknown sample’s score to those of classes which are known. Here, PLS-DA was built using spectral data from ten samples (five control, five MDX); eight LVs were used to reduce the dimensionality of the dataset. The performance of the algorithm was investigated using venetian blind cross-validation. Following this, predictions of unknowns were made using the spectral data obtained from four donors of the external validation dataset (Table 1).

### Genetic algorithm (GA)

GA was used to determine the spectral features which were the most useful for discrimination between the two classes of data. GA is a statistical technique inspired by the ideas of evolution. The algorithm aims to solve a specific problem by generating potential solutions; recombination operators are applied to the data in order to preserve critical information which can best solve the problem.^64^ Essentially, GA will identify spectral variables which provide the lowest prediction error rates, identified through a repetitive algorithm building process. In this way, it can recognize which spectral features of the dataset provide the most useful information for discriminating between different classes of data. Concurrently, it will eliminate uninformative data as well as noise from future consideration. Here, GA was applied to the training dataset which consisted of ten donors and 452 spectra. The parameters of GA are given as follows: the population size was set to 80; the mutation rate to 0.005, and the maximum number of generations for each run to 100. The breeding was fixed to double crossover, the window width was 30, and 30% of the windows were initially included. To identify the diagnostic features from within the measured Raman spectral dataset, GA was independently run 100 times which allowed for identification of significant spectral bands useful for discrimination purposes. The identified spectral features were tentatively assigned to corresponding vibrational modes, according to the literature, to determine potential biochemical basis responsible for spectroscopic differentiation (Table 2).

## Supplementary information

Supplementary information

## Data Availability

The data that support the findings of this study are available from the corresponding author upon reasonable request.
