# Supplementary material for: Diagnosis of a model of Duchenne muscular dystrophy in blood serum of mdx mice using Raman hyperspectroscopy
Source: Sci Rep. 2020 Jul 16;10:11734. doi: 10.1038/s41598-020-68598-8 (PMC7366916; doi:10.1038/s41598-020-68598-8)
Supplement: Supplementary file 1 — Supplementary information [file 41598_2020_68598_MOESM1_ESM.pdf]

Supplementary Information

Diagnosis of a model of Duchenne muscular dystrophy in blood serum of *mdx* mice using Raman hyperspectroscopy

Nicole M. Ralbovsky[a,b], Paromita Dey[b], Andrew Galfano[a], Bijan K. Dey[b,c,*], Igor K. Lednev[a,b,c*]

[a]Department of Chemistry, University at Albany, SUNY, 1400 Washington Avenue, Albany, NY 12222, USA

[b]The RNA Institute, University at Albany, SUNY, 1400 Washington Avenue, Albany, NY 12222 USA.

[c]Department of Biological Sciences, University at Albany, SUNY, 1400 Washington Avenue, Albany, NY 12222, USA

[*]Corresponding authors: Bijan K. Dey, Ph.D., email: bdey@albany.edu, and Igor K. Lednev, Ph.D., e-mail: ilednev@albany.edu

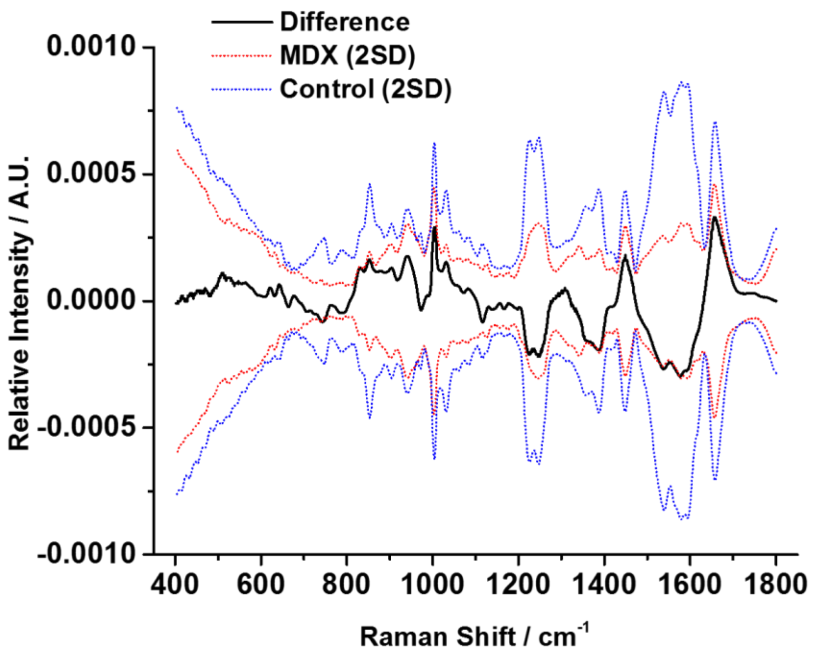

Supplementary Figure S1. The difference between the mean control and mean MDX spectra. The pre-processed difference mean blood serum spectra between Control and MDX (bold line) with ± 2 standard deviations (thin dotted lines) of the Control (blue) and MDX (red) donors' spectral data sets.
